# Polyphenols of *Camellia sinenesis *decrease mortality, hepatic injury and generation of cytokines and reactive oxygen and nitrogen species after hemorrhage/resuscitation in rats

**DOI:** 10.1186/1472-6882-10-46

**Published:** 2010-08-24

**Authors:** Mark Lehnert, Henrik Lind, Zhi Zhong, Robert Schoonhoven, Ingo Marzi, John J Lemasters

**Affiliations:** 1 Department of Pharmaceutical & Biomedical Sciences, Medical University of South Carolina, 280 Calhoun Street, Charleston, 29425, SC, USA; 2 Department of Trauma Surgery, J.W. Goethe University, Theodor Stern Kai 7, 60590 Frankfurt/Main, Germany; 3 Department of Biochemistry & Molecular Biology, Medical University of South Carolina, 280 Calhoun Street, Charleston, 29425, SC, USA; 4 Department of Environmental Sciences and Engineering, University of North Carolina at Chapel Hill, 148 Rosenau Hall, Chapel Hill, 27599 NC, USA

## Abstract

**Background:**

Reactive oxygen species (ROS) and reactive nitrogen species (RNS) are produced during hemorrhagic shock and resuscitation (H/R), which may contribute to multiple organ failure. The **Aim **of this study was to test the hypothesis that green tea (*Camellia sinenesis*) extract containing 85% polyphenols decreases injury after H/R in rats by scavenging ROS and RNS.

**Methods:**

Female Sprague Dawley rats were given 100 mg polyphenol extract/kg body weight or vehicle 2 h prior to hemorrhagic shock. H/R was induced by two protocols: 1) withdrawal of blood to a mean arterial pressure of 40 mm Hg followed by further withdrawals to decrease blood pressure progressively to 28 mm Hg over 1 h (severe), and 2) withdrawal of blood to a sustained hypotension of 40 mm Hg for 1 h (moderate). Rats were then resuscitated over 1 h with 60% of the shed blood volume plus twice the shed blood volume of lactated Ringer's solution. Serum samples were collected at 10 min and 2 h after resuscitation. At 2 or 18 h, livers were harvested for cytokine and 3-nitrotyrosine quantification, immunohistochemical detection of 4-hydroxynonenol (4-HNE) and inducible nitric oxide synthase (iNOS) protein expression.

**Results:**

After severe H/R, 18-h survival increased from 20% after vehicle to 70% after polyphenols (p < 0.05). After moderate H/R, survival was greater (80%) and not different between vehicle and polyphenols. In moderate H/R, serum alanine aminotransferase (ALT) increased at 10 min and 2 h postresuscitation to 345 and 545 IU/L, respectively. Polyphenol treatment blunted this increase to 153 and 252 IU/L at 10 min and 2 h (p < 0.01). Polyphenols also blunted increases in liver homogenates of TNFα (7.0 pg/mg with vehicle vs. 4.9 pg/mg with polyphenols, p < 0.05), IL-1β (0.80 vs. 0.37 pg/mg, p < 0.05), IL-6 (6.9 vs. 5.1 pg/mg, p < 0.05) and nitrotyrosine (1.9 pg/mg vs. 0.6 pg/mg, p < 0.05) measured 18 h after H/R. Hepatic 4-HNE immunostaining indicative of lipid peroxidation also decreased from 4.8% after vehicle to 1.5% after polyphenols (p < 0.05). By contrast, polyphenols did not block increased iNOS expression at 2 h after H/R.

**Conclusion:**

Polyphenols decrease ROS/RNS formation and are beneficial after hemorrhagic shock and resuscitation.

## Background

Patients that initially survive hemorrhage and resuscitation (H/R) may develop a systemic inflammatory response syndrome (SIRS) that leads to injury and dysfunction of vital organs (multiple organ dysfunction syndrome, MODS) [1]. Currently, only supportive therapies are available to treat SIRS and MODS [2]. The liver with its crucial involvement in metabolism and homeostasis is among the most frequently affected organs after hemorrhage-induced hypotension [3]. Systemic hypotension with resuscitation leads to complex alterations in local tissue perfusion, hypoxia and generation of reactive oxygen and nitrogen species (ROS and RNS). ROS and RNS are highly toxic metabolites that directly damage cell membranes, DNA and cell proteins. Additionally, ROS and RNS trigger release of cytokines and chemokines, leading to surface expression of adhesion molecules and leukocyte infiltration. These events produce inflammation, tissue damage and ultimately multiple organ failure [4-6]. NADPH-oxidase is an important source of superoxide radicals, and organ damage after H/R in rats diminishes with NADPH oxidase inhibitors and in NADPH-oxidase knockout mice [7,8]. Antioxidant strategies also blunt SIRS, and numerous animal studies highlight the association of a variety of inflammatory mediators with organ damage after H/R [9-13].

Plant tissues live under conditions that promote generation of reactive oxygen species, including bright light, high oxygen and heat. Chinese green tea (*Camellia sinenesis*) contains high levels of antioxidant polyphenols, including catechin, epicatechin, gallocatechin, epigallocatechin, epicatechin gallate and gallocatechin gallate [14,15]. These polyphenols are efficient free radical and singlet oxygen scavengers [12]. Accumulating data in human studies suggest that green tea may decrease the risk of cardiovascular disease and some forms of cancer. Other potential benefits include antihypertensive effects, increased bone mineral density, control of body weight and antibacterial effects, as recently reviewed [16]. In the experimental setting, beneficial effects of green tea polyphenols are also shown in ischemic brain damage, oxidant-induced cell injury and ischemia/reperfusion [17,18]. For example, polyphenol-enriched green tea extract blunts free radical generation after hepatic warm ischemia/reperfusion, as shown by electron spin resonance spectroscopy (ESR) [13]. Polyphenols also decrease graft failure after transplantation of ethanol-induced fatty livers and small-for-size rat livers, an improvement associated with a decrease of ESR detectable free radical production [19,20]. Overall, these results indicate that polyphenols exert beneficial antioxidative effects in both chronic and acute disease states. Accordingly in the present study, we evaluated the effect of plant polyphenols extracted from *C. sinenesis *on mortality and organ damage in a model of rat hemorrhage/resuscitation.

## Methods

### Hemorrhagic shock - survival model

*C. sinenesis *(green tea) extract, Sunphenon DCF-1, was obtained from Taiyo Kagaku Co., Yokkaichi, Mie, Japan, which contained 85% polyphenols by weight. Polyphenols in the extract included epigallocatechin gallate (47.2% of total polyphenols), epigallocatechin (11.0%), gallocatechin gallate (11.0%), epicatechin gallate (10.8%), gallocatechin (8.6%), epicatechin (8.4%), and catechin (3.0%) [19]. In a first set of experiments, 6 groups of 8 female Sprague Dawley rats (220-290 g) were studied: a control group in which rats were given water and 3 experimental groups in which rats received 10, 50 and 100 mg of polyphenol-enriched green tea extract/kg of body weight dissolved in 3.33 ml water/kg of body weight. *C. sinenesis *polyphenols or water vehicle were given by gavage 2 h prior to shock. After various treatments, the rats were anesthetized with pentobarbital sodium (50 mg/g body weight, i.p.). Body temperature was monitored using a telethermometer (YSI 423 s, YSI Life Sciences, Yellow Springs, OH) placed in the colon and maintained at 37°C with warming lamps. Blood pressure was monitored via polyethylene tubing (PE-50) inserted into the right femoral artery using a low pressure analyzer (LPA-200, Digi-Med, Louisville, KY). The left jugular vein was cannulated with PE-50 tubing to administer lactated Ringer's solution and to maintain anesthesia with pentobarbital whenever the corneal reflex reappeared. The right carotid artery was also cannulated with polyethylene tubing (PE-50), and shock was induced within 5 min by withdrawing blood into a heparinized syringe until mean arterial pressure decreased to 40 mm Hg. Constant pressure was maintained by withdrawal or reinfusion of small amounts of blood as necessary for 60 min.

After 60 min of hypotension, rats were resuscitated by infusion of the shed blood over 5 min. Subsequently, lactated Ringer's Solution (twice the shed blood volume) was infused over 1 h. After an additional 10 min, a blood sample for each rat was collected for determination of alanine aminotransferease (ALT). The catheters were then removed, the vessels were occluded, and the wounds were closed.

At 2 h after the end of reperfusion, blood was collected retroorbitally under anesthesia using heparinized capillary tubes for ALT measurement. At 2 or 18 h, each animal was re-anesthesized and exsanguinated. The two right-sided dorsal liver lobes were tied off and frozen in liquid nitrogen. The remaining liver was flushed by infusion of normal saline via the portal vein, followed by infusion of 4% formalin and immersion in 10% buffered formalin. The tissue was then embedded in paraffin, sectioned and stained with hematoxylin and eosin. Sham-operated animals underwent the same surgical procedures, but hemorrhage was not carried out.

### Hemorrhagic shock - non-survival model

To evaluate the effect of polyphenol extract on mortality after H/R, 2 groups of 10 female Sprague Dawley rats (220-290 g) were studied: a control group given water vehicle and an experimental group receiving 100 mg of polyphenol extract/kg, as described for the survival model. The rats were then instrumented and bled to an initial blood pressure of 40 mm Hg, as described above. Afterwards, mean arterial pressure was further progressively decreased to 28 mm Hg by withdrawal of small amounts of blood over 60 min. Resuscitation was then carried out as in the survival studies. All experiments were performed were in adherence to National Institutes of Health Guidelines for the Use of Experimental Animals using protocols approved by the Institutional Animal Care and Use Committee.

### Alanine aminotransferase

Sera were stored at -80°C for later analysis of ALT using a commercial kit (Sigma Chemical, St. Louis, MO, USA).

### Western blotting

Liver tissue was homogenized in lysis buffer at 4°C, followed by centrifugation for 30 min at 4°C at 20.000 g. Supernatants were stored at -80°C for later analysis. Lysates (50 μg protein) were separated by electrophoresis on 12% polyacrylamide SDS gels and transferred to nitrocellulose membranes (Amersham-Buchler, Braunschweig, Germany). iNOS was detected using mouse anti-iNOS antibody (Santa Cruz Biotechnology, Santa Cruz, CA, USA) Determination of β-actin with anti-β-actin antibody (Sigma, Taufkirchen, Germany) served as a loading control. Blots were blocked with 10% non-fat dry milk for 1 h, incubated 1 h at room temperature with primary antibody diluted according to the manufacturer's instructions, incubated 1 h with horseradish peroxidase-conjugated secondary antibody (Santa Cruz Biotechnology, Santa Cruz, CA, USA), and developed with ECL Western blot detection reagents (GE Healthcare, Munich, Germany).

### TNFα, IL-6, IL-1β and 3-nitrotyrosine

Frozen liver was homogenized in 25 mM Tris, 150 mM NaCl, 5 mM EDTA, 0.1% NP-40 and a cocktail of protease and phosphatase inhibitors and analyzed for TNFα, IL-6, IL-1β and 3-nitrotyrosine using commercial ELISA kits according to the manufacturers' instructions (BD Biosciences Pharmingen, San Diego, CA) [21].

### 4-Hydroxynonenal Adducts

Protein adducts of 4-hydroxynonenal (4-HNE) were detected by immunohistochemistry. Paraffin-embedded sections of liver were deparaffinized, rehydrated and incubated with a polyclonal antibody against 4-HNE (Alpha Diagnostics International, San Antonio, TX) in phosphate-buffered saline (pH 7.4) containing 1% bovine serum albumin. A peroxidase-linked secondary antibody was visualized with diaminobenzidine (Peroxidase Envision Kit, DAKO Corp., Carpinteria, CA). Sections were counter stained with hematoxylin (Richard Allan, Kalamazoo, MI). A BioQuant Nova Prime image acquisition and analysis system (BioQuant, Knoxville, TN) incorporating an Olympus BH-2 microscope (Opelco, Dulles, VA) with a QImaging digital camera was used to capture and analyze the immunostained tissue sections at 400 × magnification. The extent of labeling in the liver lobule was defined as the percentage of the field area within a preset color range determined by the software. Data from each tissue section (10 fields per section) were pooled to determine means, as described previously [8].

### Statistics

Differences between groups were determined by one-way analysis of variance (ANOVA) using a multiple comparison procedure (Tukey-test) and by the Kruskal Wallis ANOVA on ranks using a multiple comparison procedure (Dunn's method) and student's t-test. A p value of less than 0.05 was considered significant. Data shown are means ± S.E.M.

## Results

### Polyphenols improve survival after severe hemorrhagic shock and resuscitation

**Figure 1:**
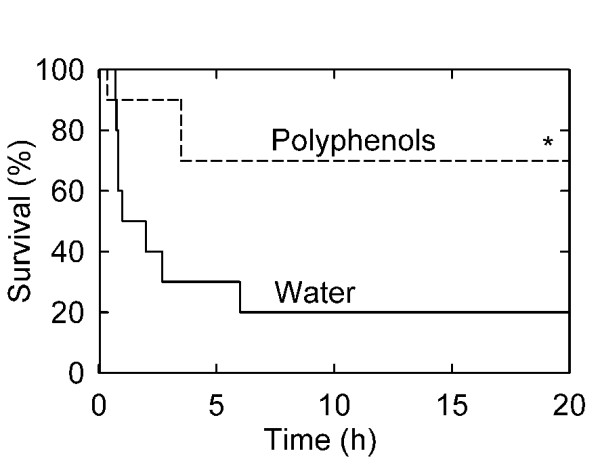
**Increased survival after hemorrhage/resuscitation in polyphenol-treated rats**. Water- and polyphenol-gavaged rats were subjected to sham operation or bled to a mean arterial pressure of 28 mm Hg and resuscitated as described in **MATERIALS AND METHODS**. Group sizes were 3-8 per group. *, p < 0.05 vs. water gavage.

Rats were gavaged with vehicle and subjected to hemorrhage to an initial arterial pressure of 40 mmHg followed by additional bleeding to decrease arterial pressure progressively to 28 mm Hg over 1 h. Resuscitation was then instituted by infusion of shed blood plus twice the shed blood volume as lactated Ringer's solution. Survival after this procedure was 20% (Fig. 1). Death occurred mainly within the first 6 h after resuscitation. In contrast, gavage of polyphenols prior to the imposition of hemorrhage improved survival to 70% (Fig. 1, p < 0.05). Polyphenol treatment did not change the amount of hemorrhage required to cause hypotension, since bleed out volume in the polyphenol-treated group was 19.4 ± 1.5 ml/kg body weight versus 21.3 ± 1.1 ml/kg body weight in the water gavage group (Table 1, p > 0.2). Blood pressure was also comparable between the polyphenol-treated and untreated hemorrhaged groups before, during and after shock (Table 1).

**Table 1:** Arterial pressures and shed blood volumes during sham operation and hemorrhage/resuscitation in the survival model.

Treatment	Water	Polyphenol	Water	Polyphenol
**Operation**	**Sham**	**Sham**	**Shock**	**Shock**

BP before hemorrhage	93 ± 1.9	92 ± 0.9	93 ± 3.4	91 ± 3.9
BP after hemorrhage			40 ± 0.1	40 ± 0.1
BP after reperfusion			96 ± 6.1	90 ± 3.8
Shed blood volume			21.3 ± 1.1	19.4 ± 1.5

Mean arterial blood pressures (BP) in mm Hg and shed blood volume in ml/kg body weight were recorded during sham operation and for different points in the hemorrhage/resuscitation protocol. Values are means ± SE.

### Polyphenols decrease release of hepatic alanine aminotransferase after moderate hemorrhage/resuscitation

**Figure 2:**
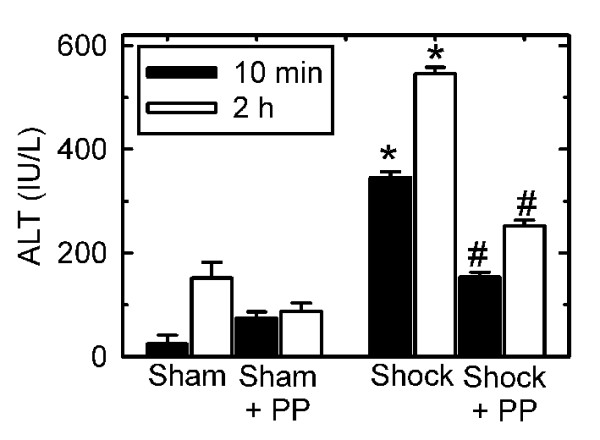
**Decreased ALT release after hemorrhage/resuscitation in rats treated with polyphenol extract**. Water- and polyphenol (PP)-gavaged rats were subjected to sham operation or bled to a mean arterial pressure of 40 mm Hg for 1 h and resuscitated, as described in **MATERIALS AND METHODS**. Blood was collected at 10 min and 2 h after resuscitation for ALT measurement. Group sizes were 4-10 per group. *, p < 0.05 vs. sham; ^#^, p < 0.05 vs. water gavage.

Rats were gavaged with vehicle and subjected to hemorrhage to a sustained hypotension of 40 mmHg for 1 h followed by resuscitation. Survival after this milder hemorrhage/resuscitation protocol averaged 80% and was not different between vehicle- and polyphenol-treated rats (data not shown). Liver injury was assessed by serum ALT at 10 min and 2 h after the end of resuscitation. In vehicle-treated rats, ALT increased to 345 ± 12 IU/l at 10 min after resuscitation compared to 24 ± 17 IU/l after sham operation (p < 0.05, Fig. 2). This finding showed liver damage beginning early after resuscitation. ALT remained elevated at 2 h after resuscitation compared to sham operation (546 ± 13 vs. 152 ± 30 IU/l respectively, p < 0.05, Fig. 2). In rats treated with polyphenols and subjected to H/R, ALT decreased by 56% after 10 min and 54% after 2 h of resuscitation compared to vehicle-gavaged rats (p < 0.05, Fig. 2). Polyphenols did not themselves affect plasma ALT, since ALT was not different in sham-operated rats gavaged with polyphenols compared to vehicle. The effect of polyphenol treatment on ALT release was dose-dependent with protection at 100 mg polyphenols/kg body weight but not at 10 or 50 mg polyphenols/kg body weight (data not shown), which was consistent with earlier studies in other models [13]. These results indicated that polyphenols at a dose of 100 mg/kg body weight substantially decreased liver damage after hemorrhage/resuscitation in rats, and this dose was used for subsequent experiments.

### Polyphenols decrease hepatic inflammatory responses after hemorrhage/resuscitation

**Figure 3:**
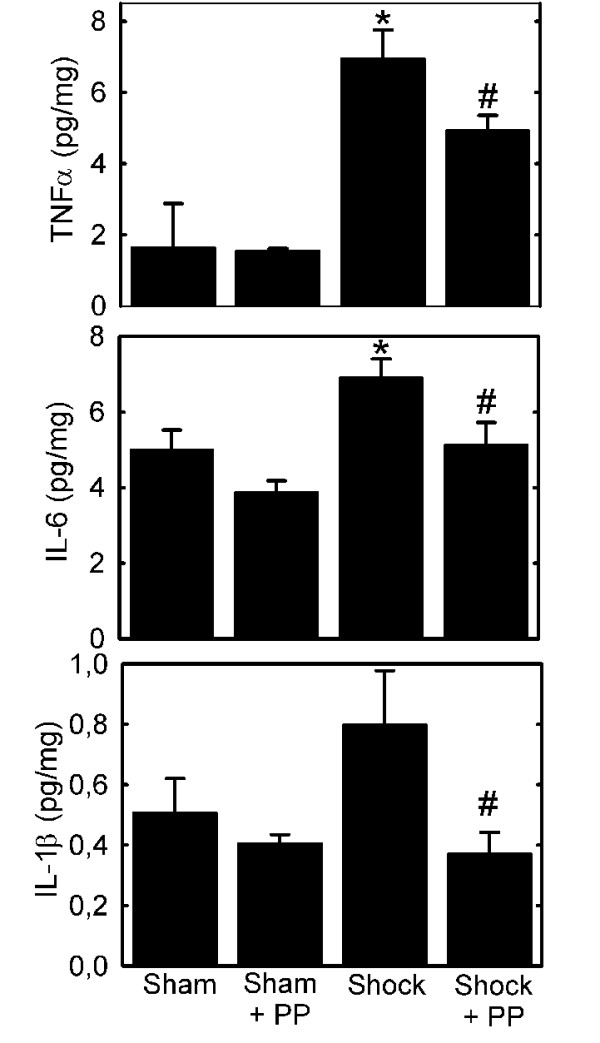
**Decreased hepatic proinflammatory cytokines in liver after hemorrhage/resuscitation in rats treated with polyphenol extract**. Water- and polyphenol (PP)-gavaged rats were subjected to hemorrhage/resuscitation or sham operation, as described in Fig. 2, and livers were harvested at 18 h after resuscitation for measurement of TNFα, IL-1β and IL-6, as described in **Materials and METHODS**. Group sizes were 4-10 per group. *, p < 0.05 vs. sham; ^#^, p < 0.05 vs. water gavage.

TNFα, IL-6 and IL-1β are proinflammatory cytokines that are implicated in organ damage after hemorrhage/resuscitation [22-24]. At 18 h after resuscitation, TNFα in liver increased more than 4-fold compared to sham operation (6.95 ± 0.8 vs. 1.64 ± 1.2 pg/mg, p < 0.05, Fig. 3). Polyphenol treatment blunted this increase (4.94 ± 0.4 pg/mg, p < 0.05, Fig. 3). Hepatic IL-6 also increased after H/R, which polyphenols also partially prevented (6.9 ± 0.48 vs. 5.1 ± 0.6 pg/mg, p < 0.05, Fig. 3). Similarly, hepatic IL-1β, an important modulator of the T-cell mediated immune response, increased 58% compared to sham operation at 18 h after resuscitation (0.80 ± 0.18 vs. 0.51 ± 0.11 pg/mg, p < 0.05, Fig. 3), and polyphenol pre-treatment blunted this IL-1β increase after resuscitation (0.37 ± 0.07 pg/mg, p < 0.05 vs. H/R) to levels comparable to sham-operated animals (Fig. 3). In sham-operated rats, polyphenol treatment did not alter hepatic levels of TNFα, IL-6 or IL-1β compared to vehicle treatment.

### Polyphenols decrease lipid peroxidation after hemorrhage/resuscitation

**Figure 4:**
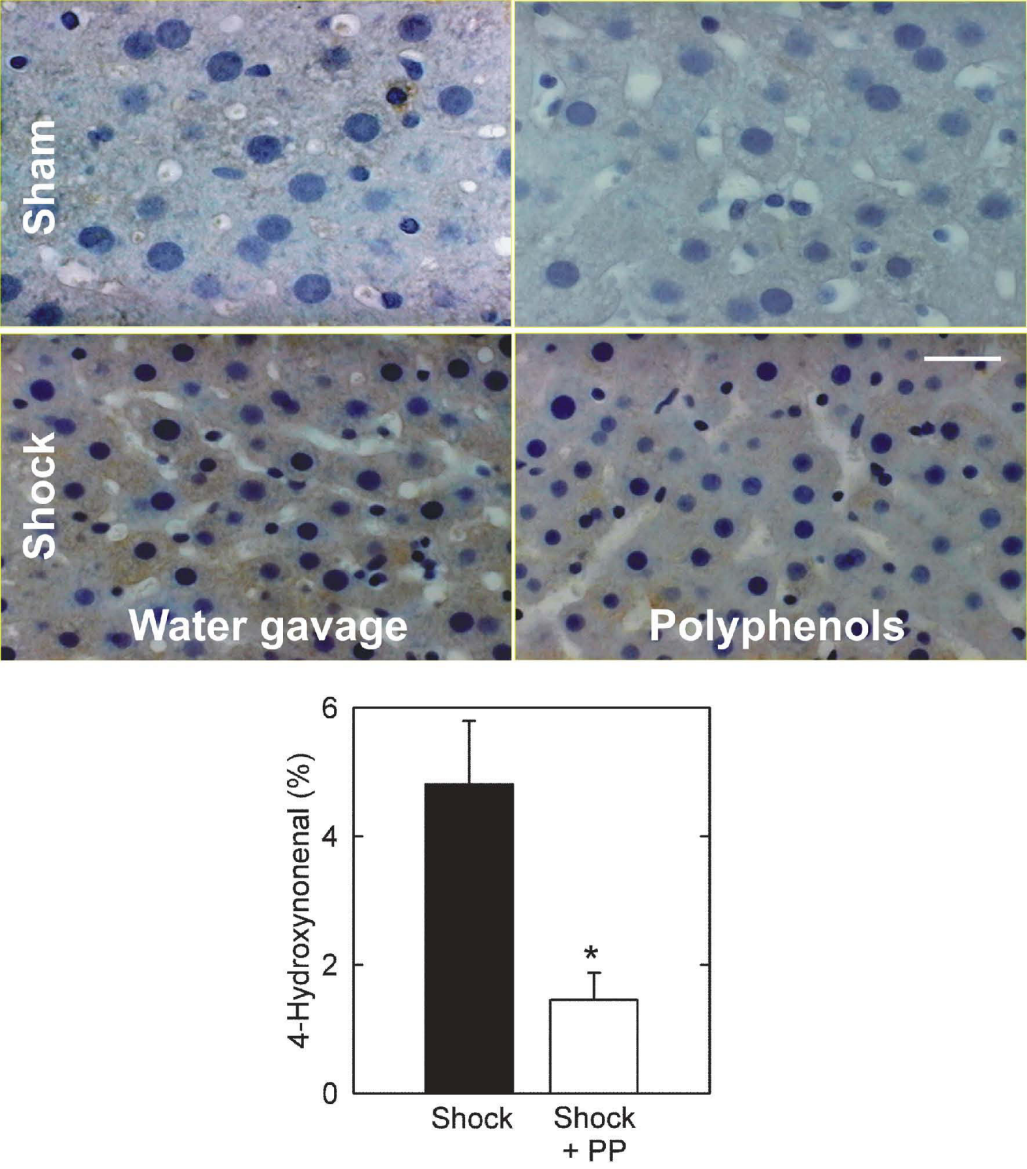
**Decreased hepatic 4-hydroxynonenal staining in rats treated with polyphenol extract after hemorrhage/resuscitation**. Water- and polyphenol (PP)-gavaged rats were subjected to hemorrhage/resuscitation or sham operation, as described in Fig. 2, and livers were harvested for immunohistochemistry at 18 h after resuscitation. 4-HNE adducts were visualized by immunocytochemistry, as described in **Materials and METHODS**. The upper panels show sections of sham-operated water-gavaged (left) and livers treated with polyphenol extract (right). The middle panels show sections of water-gavaged (left) and livers treated with polyphenol extract (right) after hemorrhage and resuscitation. The lower panel shows the percentage of 4-HNE staining, determined as described in **Materials and METHODS**. Bar is 50 μm. Group size was 6 per group. *, p < 0.05 vs wild type.

4-HNE, a product of lipid peroxidation, forms covalent adducts with proteins. Immunohistochemical detection of 4-HNE protein adducts thus serves as a marker of lipid peroxidation and oxidative stress. In livers of sham-operated rats, HNE staining was nearly undetectable, shown previously [8,19,25], but increased substantially at 18 h after hemorrhage/resuscitation (Fig. 4 and data not shown). HNE immunostaining was uniformly distributed within hepatocytes, but sinousoidal lining cells did not show evident labelling (Fig. 4). Consistent with their antioxidant properties, polyphenols decreased HNE staining by 70% (p < 0.05, Fig. 4). These results indicated that considerable lipid peroxidation occurred in liver after hemorrhage/resuscitation, which was attenuated by treatment with green tea polyphenols.

### Polyphenols decrease tyrosine nitration after hemorrhage/resuscitation

**Figure 5:**
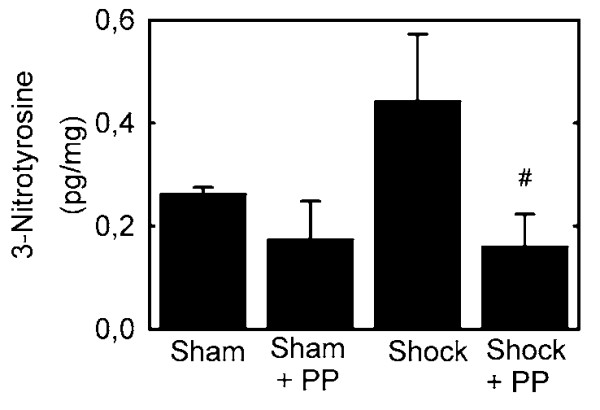
**Decreased formation of hepatic 3-nitrotyrosine adducts in rats treated with polyphenol extract after hemorrhage/resuscitation**. Water- and polyphenol (PP)-gavaged rats were subjected to hemorrhage/resuscitation or sham operation, as described in Fig. 2, and livers were harvested for measurement of 3-nitrotyrosine adducts at 18 h after resuscitation, as described in **MATERIALS AND METHODS**. Group sizes were 4-8. *, p < 0.05 vs. sham; #, p < 0.05 vs. water gavage.

Nitrotyrosine protein adducts, indicators of peroxynitrite formation and nitrative stress, form after H/R [8]. Accordingly, we evaluated hepatic protein tyrosine nitration by quantitating nitrotyrosine with an ELISA. Hemorrhage/resuscitation caused a 69% increase in hepatic nitrotyrosine content compared to sham operation. Polyphenol treatment decreased nitrotyrosine formation after hemorrhage/resuscitation (0.44 ± 0.13 vs. 0.16 ± 0.062 pg/mg, p < 0.05, Fig. 5). Nitrotyrosine levels were not different between polyphenol-treated and vehicle-treated sham-operated rats (p > 0.2). These results indicated that hemorrhage/resuscitation leads to increased peroxynitrite formation that is blocked by pretreatment with plant polyphenols.

### Polyphenols do not decrease iNOS expression after hemorrhage/resuscitation

**Figure 6:**
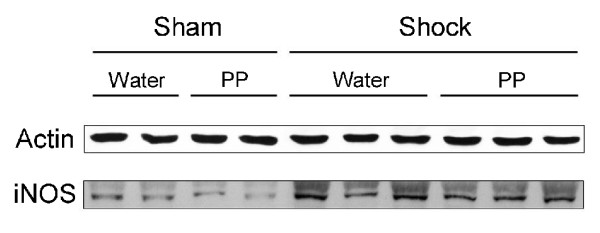
**Increased iNOS expression after hemorrhage/resuscitation - lack of protection by polyphenol pretreatment**. Water- and polyphenol (PP)-gavaged rats were subjected to hemorrhage/resuscitation or sham operation, as described in Fig. 2, and livers were harvested for measurement of iNOS protein at 2 h after resuscitation, as described in **MATERIALS AND METHODS**. A representative gel from three experiments is shown.

Previous work showed that iNOS is upregulated early after H/R and that selective iNOS inhibitors are protective [26-28]. Accordingly, we examined the effect of polyphenol treatment on hepatic iNOS protein expression at 2 h after H/R. In confirmation of the previous reports, hepatic iNOS expression increased after H/R in comparison to sham operation (Fig. 6). However, polyphenol treatment did not prevent iNOS from increasing after H/R (Fig. 6).

## Discussion

Hemorrhage/resuscitation promotes formation of reactive oxygen and nitrogen species, hepatic injury and proinflammatory cytokine release. Previous studies have shown that production of ROS and RNS increases after hemorrhage and resuscitation and that experimental strategies to scavenge free radicals decrease liver damage and prevent hepatic endothelial leukocyte adherence [7-9,29-31]. Free radical generation also increases after hepatic ischemia/reperfusion, as shown by electron spin resonance spectroscopy after both warm hepatic ischemia/reperfusion and small for size liver transplantation [13,20]. Green tea (*C. sinenesis*) extract with its high content of polyphenols inhibits lipid peroxidation *in vitro *and increases of serum antioxidative capacity *in vivo *in human subjects [11,32]. In the present study, hemorrhage/resuscitation increased hepatic 4-HNE protein adduct formation, signifying lipid peroxidation, and hepatic nitrotyrosine content, signifying peroxynitrite formation. Both changes were prevented by polyphenol treatment (Fig. 4 and 5).

Our work utilized a standardized green tea extract containing 85% total polyphenols, as measured colorimetrically. Major polyphenol species in the extract included epigallocatechin gallate, epigallocatechin, gallocatechin gallate, epicatechin gallate, gallocatechin, epicatechin, and catechin [19]. Future studies will be needed to determine which component or components are responsible for the beneficial treatment effect. Some reports indicate that the combination of polyphenols is more efficacious than a single chemically defined polyphenol, such as epicatechin [13,19,33]

Reactive oxygen species, such as hydrogen peroxide, superoxide and hydroxyl radical, promote oxidative stress *in vivo *after hepatic ischemia/reperfusion. Superoxide reduces ferric iron (Fe^3+^) to ferrous iron (Fe^2+^), which in turn reacts with hydrogen peroxide to form highly reactive hydroxyl radical (iron-catalyzed Fenton reaction) [34-36]. Additionally, superoxide reacts with nitric oxide to produce toxic peroxynitrite. Such ROS and RNS promote cell injury by damaging a variety of biologically important macromolecules, including lipids, DNA and proteins [6]. In hemorrhage/resuscitation, NADPH oxidase is a key source for superoxide production, whereas low flow states activate endothelial nitric oxide synthase (eNOS) and augment iNOS expression, resulting in increased NO production [8,37]. Consistent with these observations, our rat model of hemorrhage/resuscitation led to increased hepatic formation of both ROS and RNS, as evidenced by hepatic 4-HNE adduct formation (Fig. 4), protein nitration (Fig. 5) and increased iNOS expression (Fig 6). These events were associated with increased hepatic proinflammatory cytokine content and hepatic damage (Fig. 2 and 3). Our findings together with earlier studies illustrate the importance of increased oxidative and nitrosative stress in development of organ damage and proinflammatory changes after hemorrhage/resuscitation.

Polyphenols decreased liver injury and increased survival after hemorrhage/resuscitation. Green tea polyphenols are effective scavengers of ROS and RNS, as documented *in vitro *and *in vivo*. In the present work, polyphenols were quite effective in blunting increases of hepatic 4-HNE adduct formation and protein nitration after hemorrhage/resuscitation to rats. Polyphenols did not exert these effects by altering the hemodynamic alterations caused by hemorrhage, since shed blood volumes and arterial pressures before, during and after hemorrhage were not different between polyphenol-treated and untreated animals (Table 1). Polyphenols also did not prevent the increase of iNOS expression caused by H/R (Fig. 6). The improvement of survival (Fig. 1), decrease of hepatic injury (Fig. 2) and suppression of proinflammatory cytokine formation (Fig. 3) by polyphenols are most likely the consequence of ROS and RNS scavenging. Recent studies from this laboratory also show that green tea polyphenols decrease liver injury after warm hepatic ischemia/reperfusion and after transplantation of reduced size livers and ethanol-induced fatty livers [13,19,20]. Although, the molecular mechanism of polyphenol cytoprotection remains to be determined exactly, our data are consistent with the conclusion that quenching of toxic superoxide and/or peroxynitrite by polyphenols is responsible for cytoprotection. Indeed, since superoxide is required for peroxynitrite formation from NO, superoxide quenching alone might be sufficient for cyoprotection. Taken together, these findings show that green tea polyphenols are potentially an effective therapy in diseases where liver ischemia/reperfusion plays a pathogenic role.

In our non-survival model of hemorrhage/resuscitation, polyphenol treatment improved survival from 20% to 70% (Fig. 1). Consistent with our results, previous studies show that hypotension to 30 mm Hg or less for 1 h or longer leads to mortality in rats [38]. Mortality after hemorrhagic shock has been attributed, at least in part, to TNFα production [24,38]. Antibodies to TNFα decrease mortality and revert hyporeactivity to epinephrine in isolated rat aortic rings, a surrogate parameter for vasoplegia after hemorrhage [24]. Additionally, recombinant TNF-binding protein decreases rolling adhesion and firm adhesion of leukocytes to the hepatic interstitium after hemorrhage [24,39]. Similarly, increased IL-1β in liver and other organs is associated with hepatic injury, renal dysfunction and pulmonary leukocyte infiltration after H/R [22,40,41]. IL-6 is also associated with organ damage in various hemorrhage models (Fig. 3) [23,42]. Monoclonal IL-6 antibody protects against trauma-hemorrhage induced cardiac dysfunction, hepatic dysfunction and liver injury, and IL-6 knockout mice are protected from postresuscitation inflammation after hemorrhage [43]. By contrast, IL-6 decreases LPS-induced mortality in mice and LPS-induced TNFα release by human monocytes [44,45]. In our experiments, radical-scavenging plant polyphenols blunted TNFα, IL-1β and IL-6 production after hemorrhage/resuscitation, indicating that ROS/RNS contribute to cytokine formation and release.

## Conclusion

Taken together, our data are consistent with the conclusion that mortality, liver injury and hepatic proinflammatory changes were consequences of evolving oxidative and nitrosative stress during hemorrhage/resuscitation. Our work is clinically relevant for ischemia/reperfusion in a planned setting such as cardiac surgery; however, future studies will be needed to address whether polyphenols can be protective in a rescue paradigm. Overall, plant polyphenols as potent scavengers of ROS and RNS represent a potential therapy to prevent liver injury and proinflammatory changes associated with resuscitated blood loss.

## Competing interests

This work was supported, in part, by Fresenius Kabi Deutschland GmbH.

## Authors' contributions

ML participated in the design of the study, set up the hemorrhage model and drafted the manuscript. HL performed the hemorrhage experiments, including polyphenol feeding. ZZ helped with the statistical analysis and interpretation of the data. RS performed immunostaining and analysis. IM participated in conceiving and coordinating the experiments. JJL had responsibility for overall planning and conduct of the work and final editing of the manuscript. All authors read and approved the final manuscript.

## Pre-publication history

The pre-publication history for this paper can be accessed here:

http://www.biomedcentral.com/1472-6882/10/46/prepub
